# Supplementary material for: Artificial Intelligence Techniques Used to Extract Relevant Information from Complex Social Networks
Source: Entropy (Basel). 2023 Mar 16;25(3):507. doi: 10.3390/e25030507 (PMC10048000; doi:10.3390/e25030507)
Supplement: Supplementary file 1 [file entropy-25-00507-s001.zip › entropy-2213641-supplementary.pdf]

Supplementary Material

Artificial intelligence techniques used to extract relevant information from complex social networks

Santiago Paramés-Estévez[1,2], Alejandro Carballosa[1,2], David Garcia-Selfa[1,2,3] and Alberto P. Munuzuri[1,2*]

[1] Group of NonLinear Physics, Univ. of Santiago de Compostela, 15706 Santiago de Compostela (Spain)

[2] Galician Center for Mathematical Research and Technology (CITMAga), 15782 Santiago de Compostela, Spain

[3] CESGA (Supercomputing Center of Galicia), Avda. de Vigo s/n, Santiago de Compostela, 15705, Spain

[*] Correspondence: alberto.perez.munuzuri@usc.es

S1.- Examples of hashtags used to build up the networks.

In this section, we present some examples of the hashtags considered in order to build up the networks. Only five days are presented in Table S1 although an equivalent set of hashtags was used for each of the days analyzed.

To prevent adding any bias, topics were chosen considering their popularity in the social network, that is, the number of users involved. For each day a ranking is stablished and the six more popular are presented for a selection of days. Mar. 15, 2020 was a critical date when mandatory confinement was ordered in Spain in order to control the COVID-19 pandemic expansion. Note that some of the dates in the table are from before (marked in light green), right after the confinement started (marked in red) and several months after (marked in light yellow).

Table S1: Examples of the hashtags used to build up networks each day. Hashtags are ordered following the popularity of the topic (most popular on top).

Date	Top Hashtags		# Nodes	# Links
2020/01/25	1	#OTDirecto25E	11940	8058
	2	#TdelDescuento25E	9223	7602
	3	#Goya2020	1139	608
	4	#COAC2020P5	885	526
	5	#TeQueremosCholo	1690	1084
	6	#MCJunior	2910	1952
2020/02/1	1	#OTDirecto1F	12378	8790
	2	#HappyBirthdayHarry	9044	6102
	3	#TdelDescuento1F	784	597
	4	#La Gomera	18171	9652
	5	#TCMS4	2143	1545
	6	#COAC2020P12	481	274
2020/03/1	1	#Beauxbatons	3570	1917
	2	#FelizDomingo	15960	8680
	3	#l6ncoronavirus	5995	3340
	4	#poligianadara	11235	8748
	5	#Prodigios3	204	136
	6	#AnimalCrossingConTerry	416	246
2020/03/16	1	#DirectoAcademia16M	11805	8517
	2	#covid19ESP	17813	9772
	3	#ConexionHonduras4	8098	5844
	4	#NO VALE MENTIR	414	215
	5	#Margarita Robles	13841	7823
	6	#Alfred	6933	4430
2020/05/3	1	#AssassinsCreedValhalla	14482	8689
	2	#NoAlEstadoDeAlarma	15492	9075
	3	#Ifema	16568	9550
	4	#COVID__19	17826	9628
	5	#polimerlosplace	435	255
	6	#FelizDiadeLaMadre	18493	9993
2020/06/10	1	#CanalOT10J	14088	9433
	2	#Ayax	14664	8030
	3	#Miguel Bosé	18753	9832
	4	#Avispa	2032	1135
	5	#Tolkien	17977	9874
	6	#Casablanca	843	437

S2.- Graph construction from hashtags.

Using freely available information from *Twitter*, we searched for the first five key words or hashtags that had been trend in Spain daily, and then used our software to collect at least 10000 interactions per topic. Then, merging the datasets obtained from the hashtags we built a network for each day where users were placed as nodes and interactions as directed links. The process to join the networks from different hashtags is shown in Figure S1. Here each panel show the location of the nodes introduced when considering that particular hashtag till the whole network was reconstructed (Figure S1f).

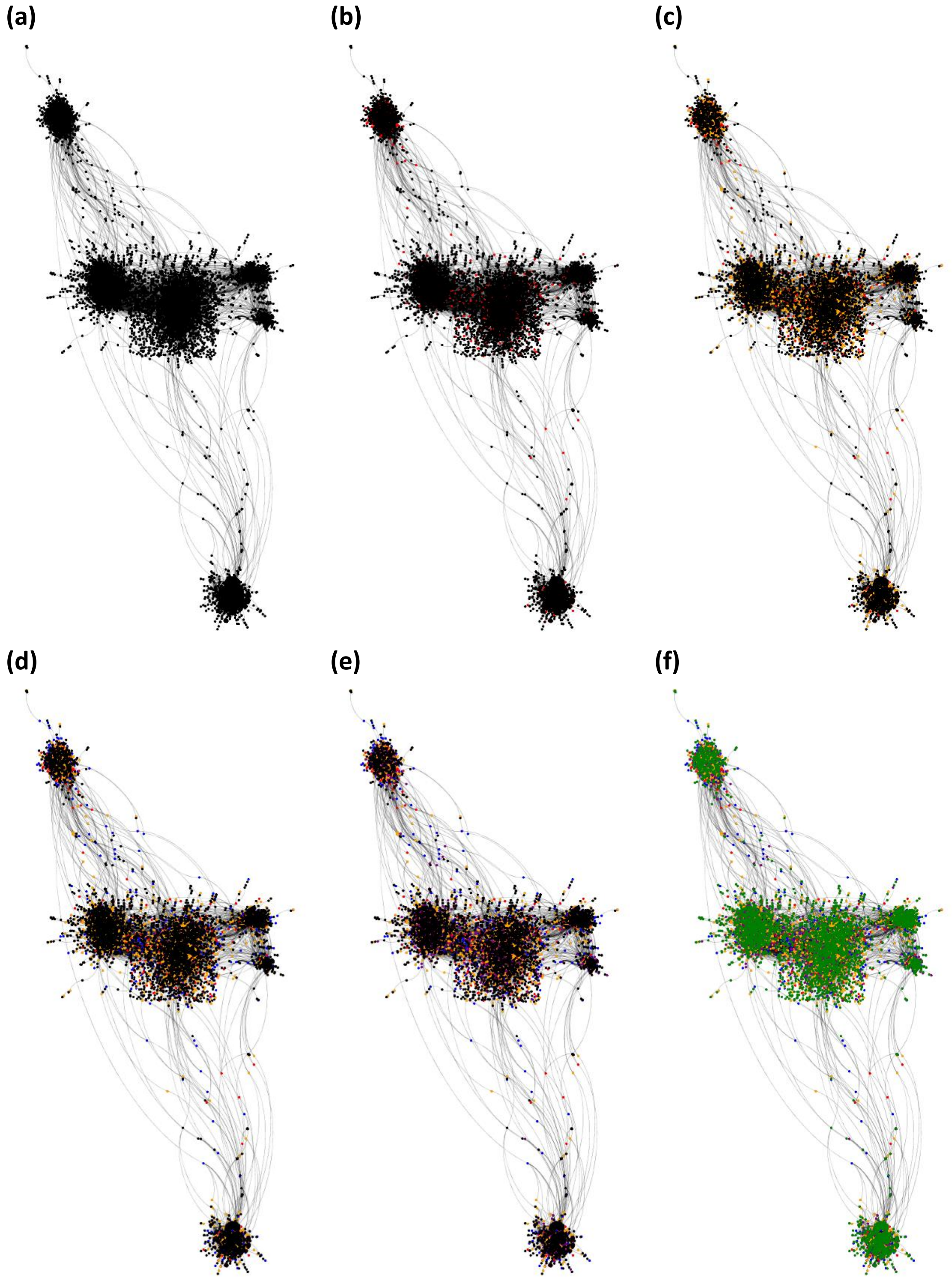

Figure S1: Users interactions in the social network are unveiled analyzing several hashtags. The addition of several networks resulting from several hashtags analysis is shown in this sequence of images that include additional hashtags, with the color representing which theme they interact mostly with. In each panel, a new hashtag is added progressively till the whole network was reconstructed in (f).

S3.- Diagram of the architecture of our convolutional neural network.

Figure S2 shows the CNN architectures used to calculate the structural parameters of each network. Note that two architectures are presented. The first one describes the architecture used to calculate the scaling exponents. The second describes the CNN used to calculate the average shortest path length ℓ and the network diameter $\overline{\ell_{\max}}$.

The performance of any model is highly dependent on the type of data it is trained on. Both models were tried for all four parameters analyzed along the text (γ_{in}, γ_{out}, $\overline{\ell}$ and $\overline{\ell_{max}}$) and the architecture with the best fit to the data was chosen as the most appropriate model in terms of accuracy in the predictions.

Since the objective was to prove the possibility to estimate parameters with only images of the networks while keeping the model as simple as possible, only slight adjustments in the number of convolutional and pooling layers are considered to improve the observed results.

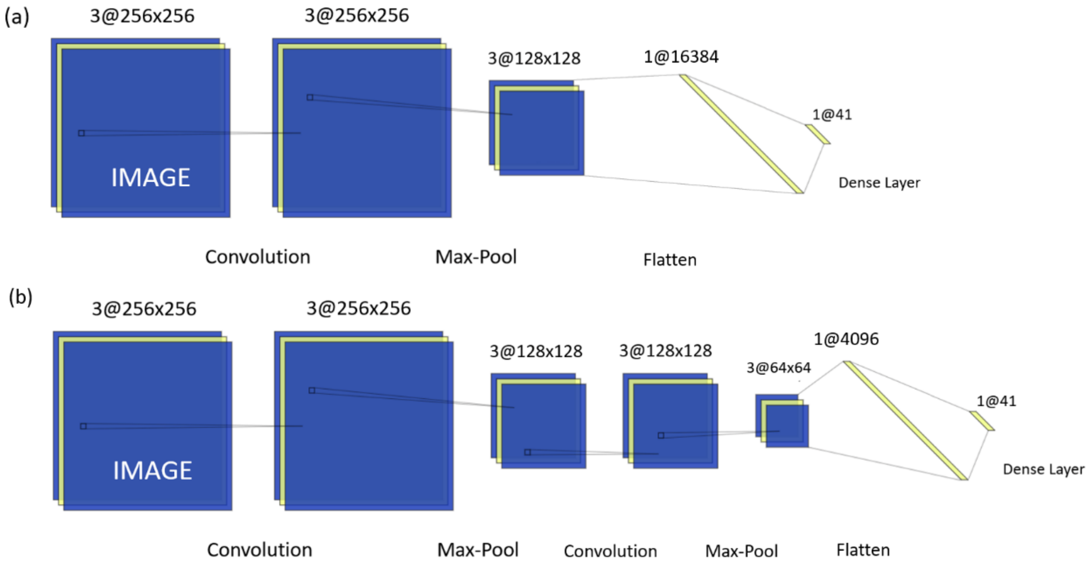

Figure S2: Both architectures used in this work: (a) works better for $\overline{\ell}$ and $\overline{\ell_{max}}$ and (b) for γ_{in} and γ_{out}. Convolutional and Max-Pooling layers are applied alternatively to the original images, converting their values in final vector which the parameter values are extracted from.

S4.- Temporal evolution of the morphological parameters calculated.

Evolution of the calculated parameters that characterize the network. The networks cover a temporal interval from June 2019 till March 2021. Each network had more than 10000 nodes and the main features can be described by this set of parameters.

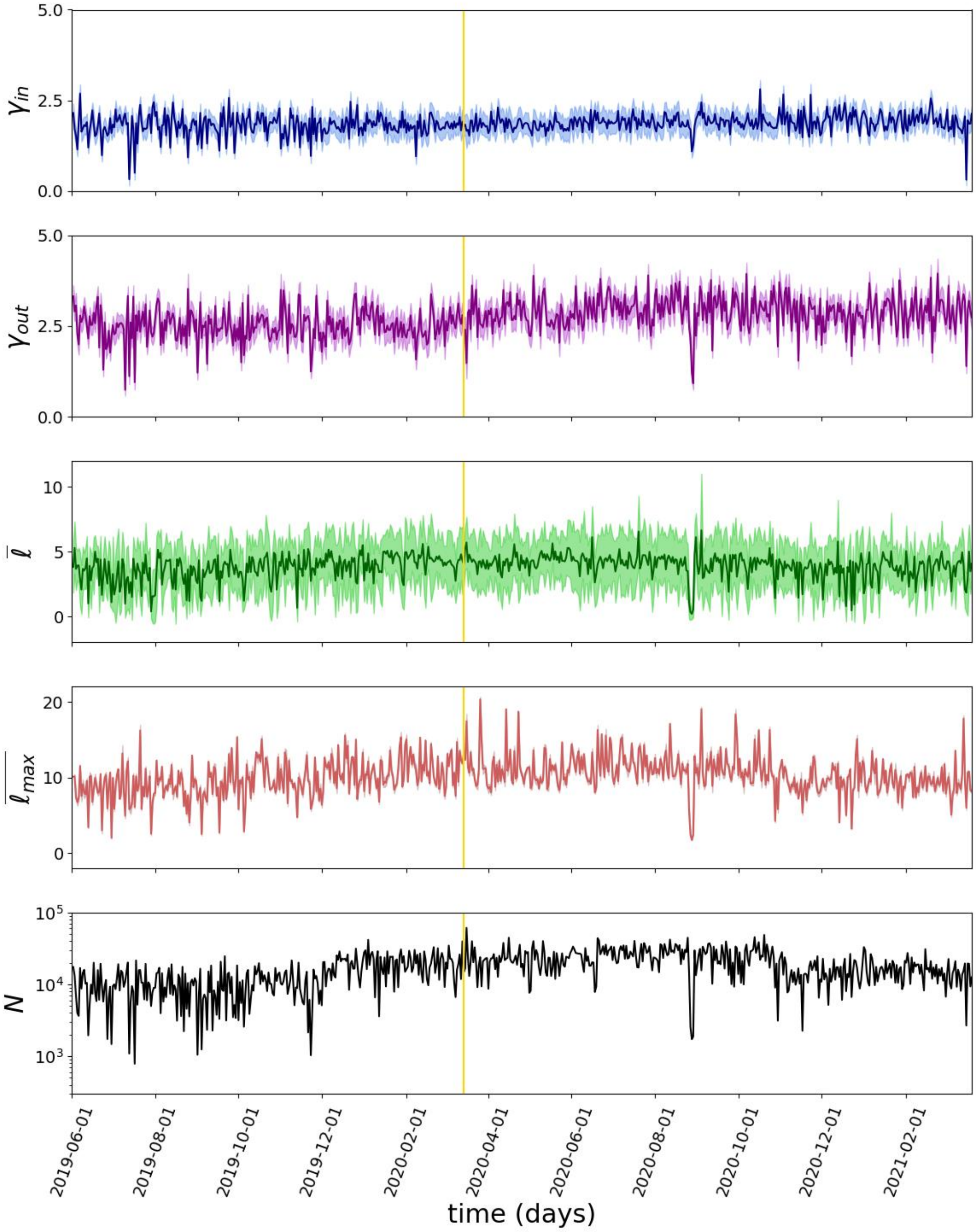

Figure S3. Time evolution of the scale-free exponent γ_{in} (a), γ_{out} (b), the average shortest path length ℓ (c), the network diameter $\overline{\ell_{max}}$ (d) and the number of nodes per network N (e). The yellow line denotes the start of the mandatory confinement ordered by the government in Spain due to the COVID-19 pandemic.

S5.- Examples of plots with different networks.

Figure S4 shows several examples of the images created from the data downloaded from *Twitter* in a single day and showing the network configuration. The networks are constructed following the protocol described in the Methods section in the main text. These images were used to train the CNN that was later used to rapidly calculate the set of parameters characterizing our networks.

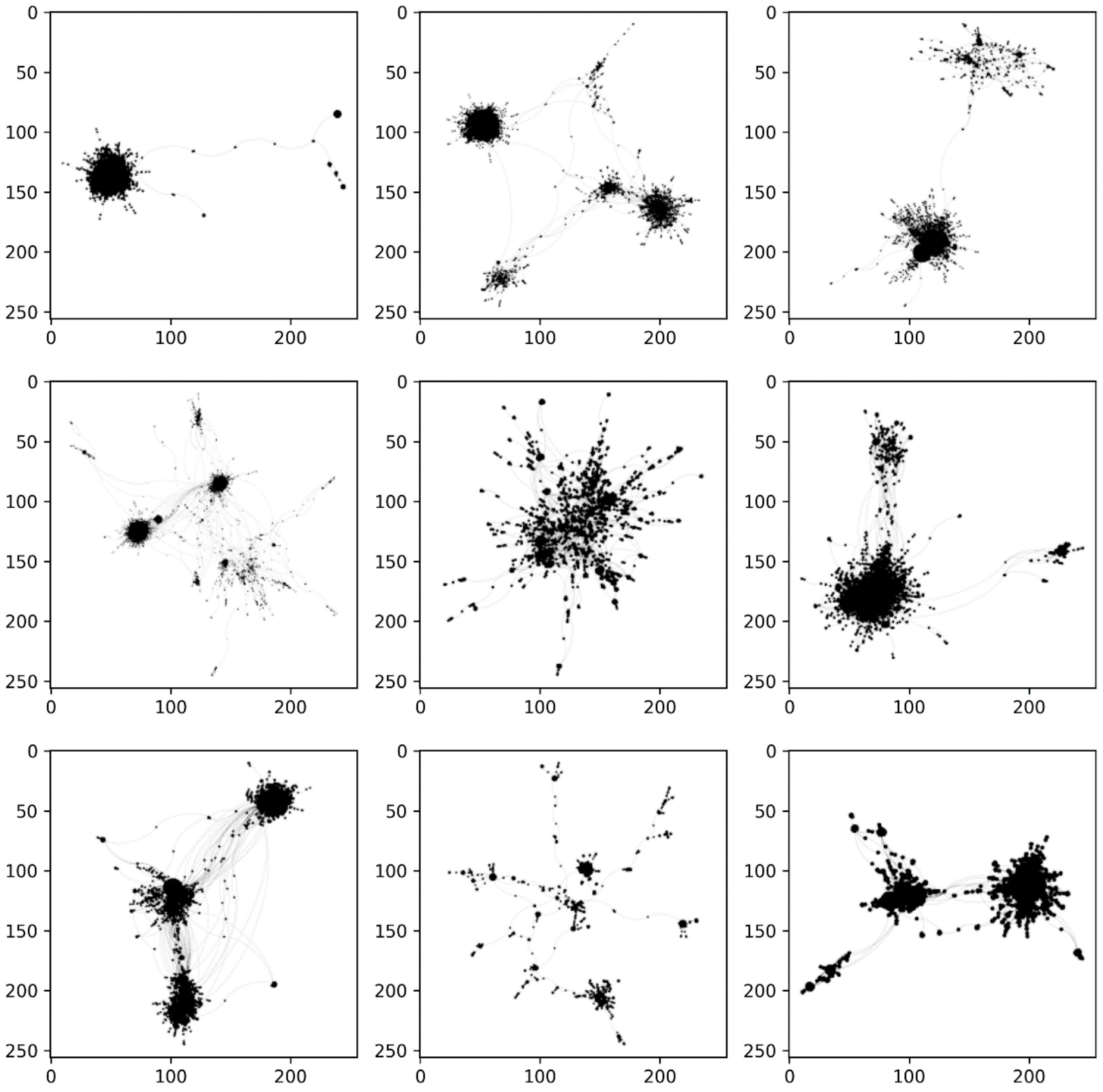

Figure S4. Samples of the data fed to the neural network.

S6.- Animations for the Supplementary material.

Two animations are included as separate files that illustrate the evolution of the networks as more hashtags are included:

- Repulsion.gif shows the structure of the network are plotted using Gephi as more hashtags are added.

- AddingHashtags.gif plots with different colors, the nodes corresponding with the different hashtags as they are being added. The structure of the network (in black) is the final structure considering all hashtags included.

Images were made in Gephi with the Force Atlas 2 algorithm with scaling 0.3 and gravity 2.0, the rest of the parameters were left in their default values.

(a) (b)

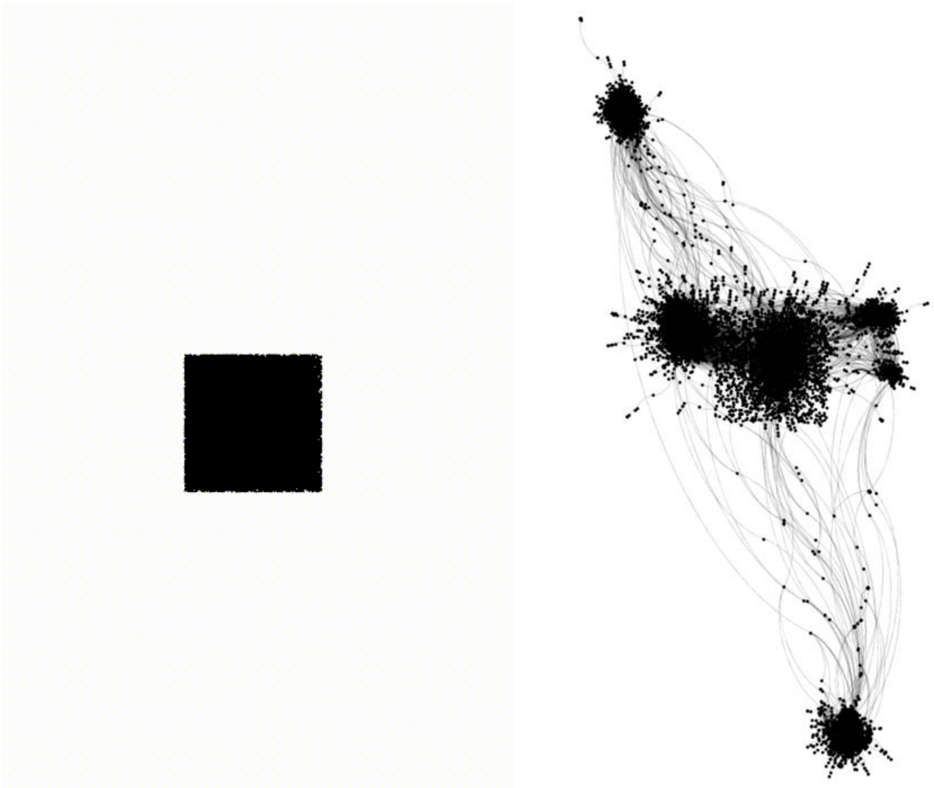

Figure S5. Images are standardized with a repulsion and attraction algorithm in Gephi, a complex network representation software, this process has been animated in (a). Users in the network interact with one another using different hashtags, represented in (b) by colors (each color indicates the hashtag where that particular node was more active).

S7.- Cost functions for the CNN used.

Figure S6 shows the convergence of the observed losses for each of the models used for each parameter. Figures S6a and b appear to converge somewhat faster than the other two. Again, our objective in this work is to show that this type of neural network is a tool that has this capability, even with a very basic architecture, with hardly any optimization of its hyperparameters. Of course (but this is beyond the scope of this paper), with optimized and more conscientious training, values could be predicted much more accurately.

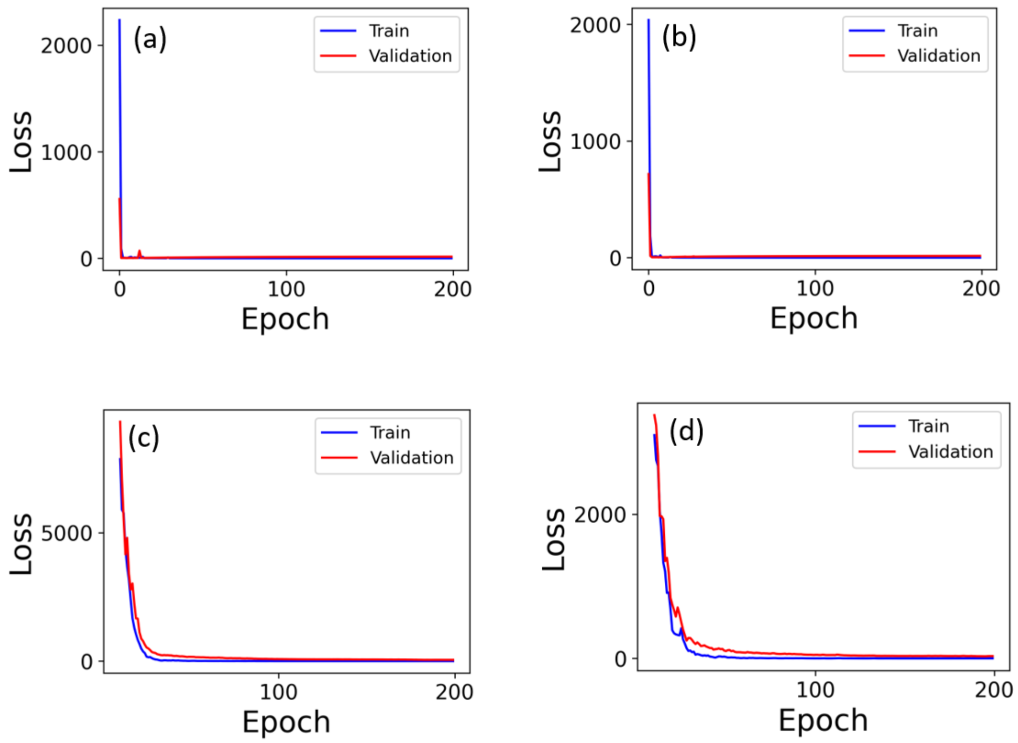

Figure S6: Mean squared error losses for each epoch and for each one of the four models: (a) γ_{in}, (b) γ_{out}, (c) $\overline{\ell}$ and (d) $\overline{\ell_{\max}}$. Panels (c) and (d) show the data starting from the 10th epoch.
